# Supplementary material for: Spectroscopy Approach for Highly-Efficient Screening of Lectin-Ligand Interactions in Application for Mannose Receptor and Molecular Containers for Antibacterial Drugs
Source: Pharmaceuticals (Basel). 2022 May 19;15(5):625. doi: 10.3390/ph15050625 (PMC9146875; doi:10.3390/ph15050625)
Supplement: Supplementary file 1 [file pharmaceuticals-15-00625-s001.zip › pharmaceuticals-1698095-supplementary.pdf]

- Figure S1: Scheme of synthesis of mannosylated HPCD-spermine-Man-**1** and HPCD-spermine-Man-**2** conjugates. 2-hydroxypropyl-β-cyclodextrin (HPCD) diagrammatically depicted in the form of a heptagon. The characteristics of the synthesized ligand are given in Table 1;
- Figure S2: Scheme of synthesis of mannosylated HPCD-spermine-Man-**3** conjugate. 2-hydroxypropyl-β-cyclodextrin (HPCD) diagrammatically depicted in the form of a heptagon. The characteristics of the synthesized ligand are given in Table 1;
- Figure S3: Scheme of synthesis of mannosylated HPCD-PEI35-Man-**4** conjugate. On average, two hydroxypropyl groups are activated in HPCD-CDI. The characteristics of the synthesized ligand are given in Table 1;
- Figure S4: Scheme of synthesis of mannosylated HPCD-PEI35-Man-**5** conjugate. On average, seven hydroxypropyl groups are activated in HPCD-CDI. The characteristics of the synthesized ligand are given in Table 1;
- Figure S5: Scheme of synthesis of mannosylated HPCD-PEI35-Man-**6** conjugate. The characteristics of the synthesized ligand are given in Table 1;
- Figure S6: Scheme of synthesis of mannosylated HPCD-PEI1.8-Man-**8** conjugate. The characteristics of the synthesized ligand are given in Table 1. On average, one cyclodextrin torus accounts for 4-7 branched mannosylated chains of PEI;
- Figure S7: Decomposition of the IR spectrum of the HPCD-spermine-Man-**1** conjugate in the 980-1130 cm^{-1} region into HPCD and Man components. The fitted spectrum is given;
- Figure S8: Fourier transform infrared spectra of mannosylated spermine-series conjugates: (a) HPCD-spermine-Man-**1**, (b) HPCD-spermine-Man-**2**, (c) HPCD-spermine-Man-**3**;
- Figure S9: Fourier transform infrared spectra of mannosylated PEI35-series conjugates: (a) HPCD-PEI35-Man-4, (b) HPCD-PEI35-Man-5, (c) HPCD-PEI35-Man-6. (d) 1H NMR spectrum of HPCD-PEI35-Man-6. D_2O, 12 mg/ml, 400 MHz;
- Figure S10: Fourier transform infrared spectra of mannosylated branched-PEI-series conjugates: (a) HPCD-PEI1.8-Man-**8**, (b) HPCD-PEI10-Man-**9.** ^{1}H NMR spectra of conjugates: (c) HPCD-spermine-Man-**7**, (d) HPCD-PEI1.8-Man-**8**, (e) HPCD-PEI10-Man-**9**. D_2O, 12 mg/ml, 400 MHz;
- Figure S11: A fluorescent approach to studying the interaction of FITC-labeled cyclodextrin with ConA. Titration of a FITC-labeled ligand solution (C_{FITC} ≈ 0.2 µM) in 550 µl Tris/HCl buffer solution (C = 0.01 M, pH = 7.2) containing 0.5 M NaCl, 1 mM $CaCl_2$, 1 mM $MnCl_2$ was performed by adding aliquot ConA (10-25 mg/ml) 2-200 µl at 22 °C;
- Figure S12: Stern-Vollmer's fitting plot of fluorescence intensity in case competitor binding of HPCD-PEI35-Man-**5** conjugate with ConA in mannose solution [Man] = 0.5 M. [L] – the equilibrium concentration of the ligand. C_0(dimeric ConA) = 38 µM. Ligand concentration varied from 1 to 250 µM. Natrium-citric buffer solution (0.04 M, pH 5.5). $C(Ca^{2+}) = C(Mn^{2+})$ = 1 mM. T = 22°C;

- Figure S13: Tryptophan fluorescence spectra: titration of ConA by mannan. Titration of a dimeric ConA solution (C = 19 μM) in 500 μl of a sodium citrate buffer solution (C = 0.02 M, pH 5.5) containing 0.5 M NaCl, 1 mM $CaCl_2$, 1 mM $MnCl_2$ was carried out by adding aliquot of mannan at 22 °C. Concentration of mannan varied in row 0; 1.3 μM; 3.9 μM; 8 μM; 26 μM to 78 μM;
- Figure S14: Complex ConA–trimannoside: X-ray diffraction data of PDB (a) and after 50-ns molecular dynamics simulation (b). The green sphere is Ca^{2+}. From [17];
- Figure S15: Maximum position in Amide II areas in differential IR spectra of the second order. Normalized to the amide I band Fourier transform infrared spectra of ConA: titration of ConA by ligand HPCD-spermine-Man-**2** and **3**. C_0(ConA) = 33 μM. Natrium-citric buffer solution (0.04 M, pH 5.5). C(Ca^{2+}) = C(Mn^{2+}) = 1 mM. T = 22°C;
- Figure S16: Hill's linearization of hyperbolic curves of dependences of maximum position in differential IR spectra of the second order (as in Fig. S15). The conditions are similar to those specified in Fig. S15. [L] – the equilibrium concentration of the ligand. Theta – percentage of occupied binding sites. lg K_d value is calculated as the ratio of the length of the segment cut off on the ordinate axis to the value of the tangent of the angle of inclination;
- Figure S17: A fluorescent approach to studying the interaction of levofloxacin (Lev) with mesh HPCD-spermine-Man-**2** and polymer HPCD-PEI35-Man-**4** conjugates. Quenching and anisotropy of Lev fluorescence: λ_{exci} = 396 nm, λ_{emi} = 480 nm. Titration of Lev solution (C_0 = 5 mg/ml, 1 mM HCl) by ligands. T = 22°C;
- Figure S18: FTIR spectra of Lev (C0(Lev) = 5 mg/ml) in the absence of spermine (blue spectrum) and in the presence of spermine (concentration gradient from red to green – from 2 to 5 mM). 10 mM HCl solution. T = 22°C;
- Figure S19: The dependence of the peak intensity at 1474 cm^{-1} in the IR spectra of levofloxacin complexes with conjugates normalized for the range of 2800-1800 cm^{-1}. The insert shows the normalized IR spectra for HPCD-spermine-Man-**2** complexes with Lev. C_0(Lev) = 5 mg/ml. 1 mM HCl solution. T = 22°C;
- Figure S20: Solid phase FTIR spectra of levofloxacin (Lev), Lev–HPCD complex, and Lev complex with HPCD-PEI35-Man-**5** conjugate;
- Figure S21: Solid phase FTIR spectra of eugenol, eugenol–HPCD complex, and dual (Lev ang eugenol) drug inclusion complexes with HPCD-PEI35-Man-**5** conjugate;
- Figure S22: (a) Integral intensity of 1184–1680 cm^{-1} region in solid phase FTIR spectra of levofloxacin (Lev) and eugenol (EG) double complex with HPCD-PEI35-Man-**5** conjugate, superimposed on microphotography samples of 1x1 microns in size. (b) Corresponding FTIR spectra and areas on the micrograph (c);

- Figure S23: Result of deconvolution of the absorption aromatic band at 1495–1415 cm^{-1} in the FTIR spectra for the (a) Lev, and (b) Lev – HPCD-PEI35-Man-**5** complex;
- Figure S24: High-performance liquid chromatogram of conjugates HPCD-spernine-Man-7 (5 mg/ml) and HPCD-PEI1.8-Man-8 (two portions, 10 mg/ml). UV-absorption detector (204 nm). The eluent was 15 mM PBS (pH 7.4) containing 150 mM NaCl; the elution rate was 0.5 mL/min, 25°C;
- Figure S25: (a) Spectrophotometric titration of amino groups (before and after mannosylation) with 2,4,6-trinitrobenzenesulfonic acid for conjugate HPCD-spermine-Man-**7**. ε(2,4,6-trinitrobenzenesulfonic acid with NH_2-group adduct) = $1.3 \cdot 10^4$ $M^{-1} \cdot cm^{-1}$. (b) Calibration dependence of the fluorescence intensity of the reaction product on the concentration of primary amino groups (standard – spermine). Fluorescence analysis with *ortho*-phthalic aldehyde and 2-mercaptoethanol (1 minute for incubation). T = 22°C.

Figure S1. Scheme of synthesis of mannosylated HPCD-spermine-Man-**1** and HPCD-spermine-Man-**2** conjugates. 2-hydroxypropyl-β-cyclodextrin (HPCD) diagrammatically depicted in the form of a heptagon. The characteristics of the synthesized ligand are given in Table 1.

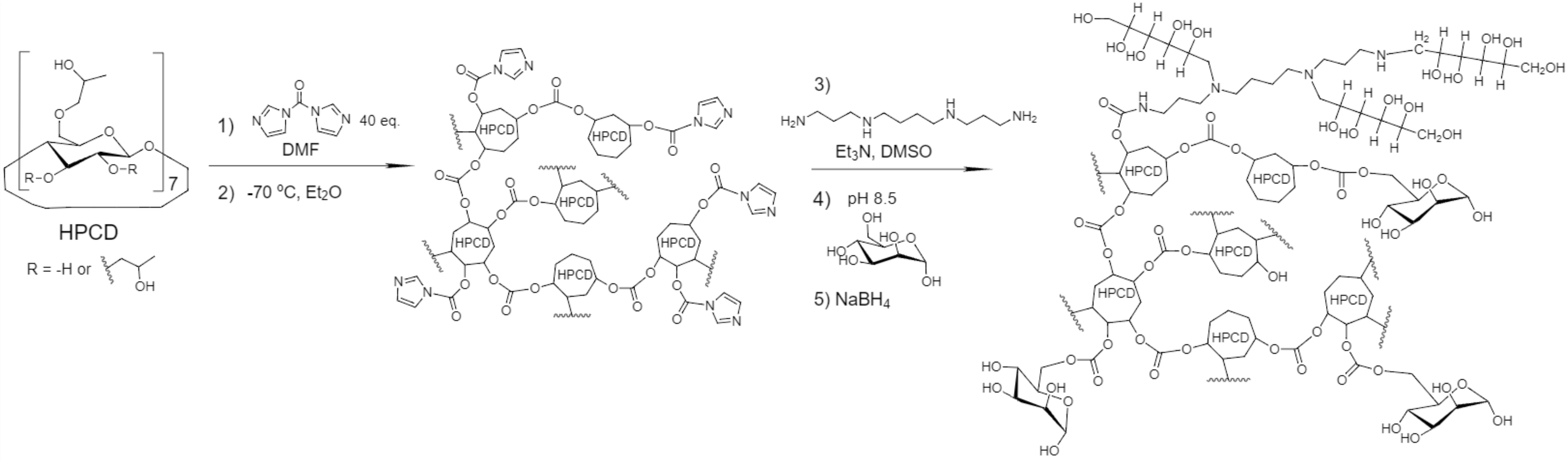

Figure S2. Scheme of synthesis of mannosylated HPCD-spermine-Man-**3** conjugate. 2-hydroxypropyl-β-cyclodextrin (HPCD) diagrammatically depicted in the form of a heptagon. The characteristics of the synthesized ligand are given in Table 1.

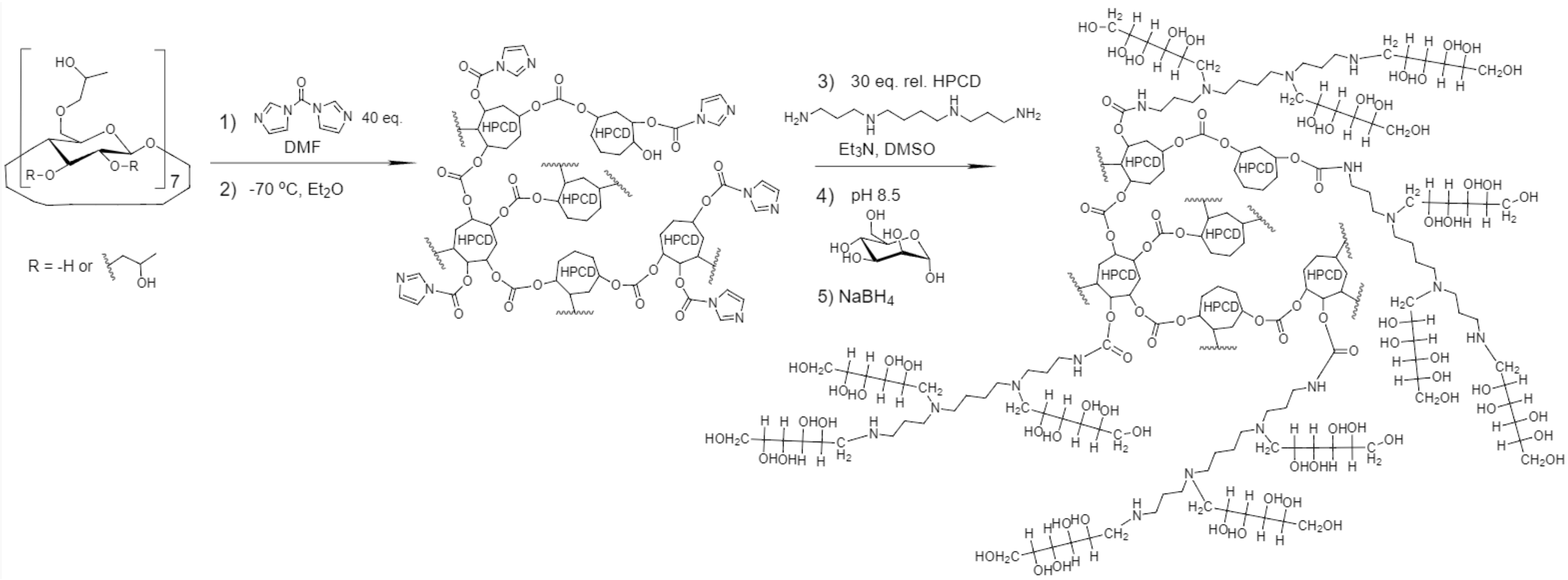

Figure S3. Scheme of synthesis of mannosylated HPCD-PEI35-Man-**4** conjugate. On average, two hydroxypropyl groups are activated in HPCD-CDI. The characteristics of the synthesized ligand are given in Table 1.

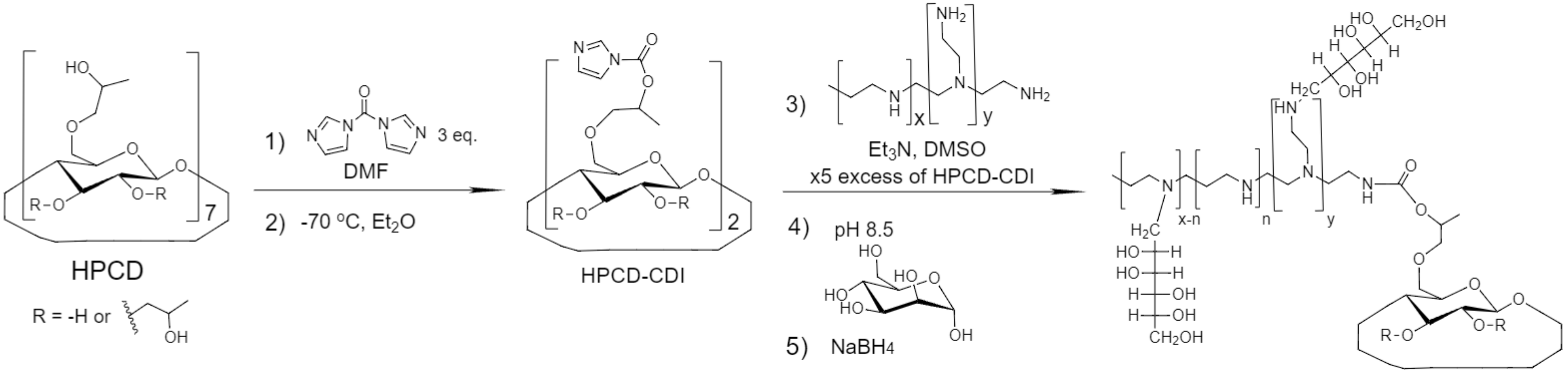

Figure S4. Scheme of synthesis of mannosylated HPCD-PEI35-Man-**5** conjugate. On average, seven hydroxypropyl groups are activated in HPCD-CDI. The characteristics of the synthesized ligand are given in Table 1.

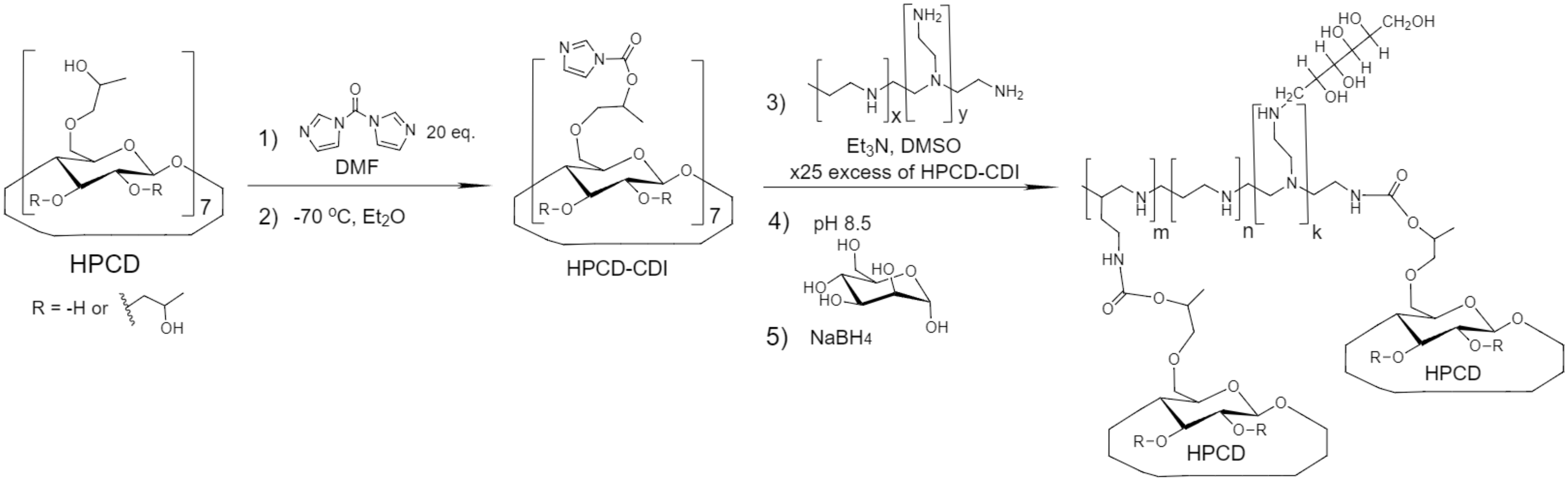

Figure S5. Scheme of synthesis of mannosylated HPCD-PEI35-Man-**6** conjugate. The characteristics of the synthesized ligand are given in Table 1.

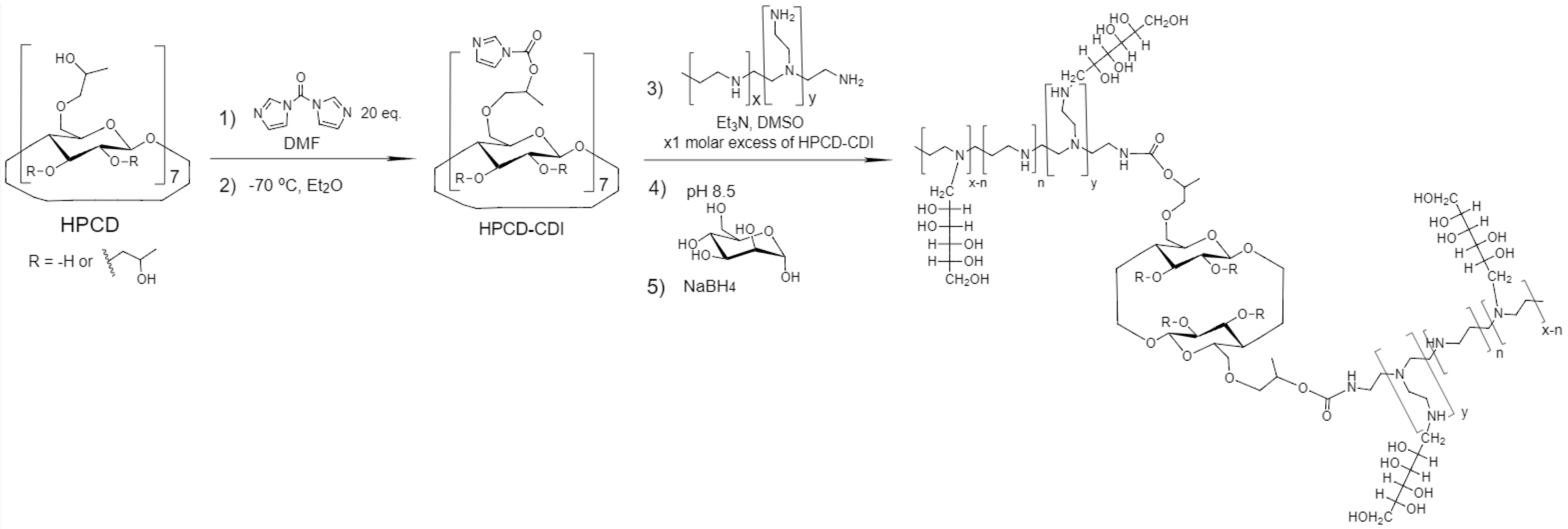

Figure S6. Scheme of synthesis of mannosylated HPCD-PEI1.8-Man-**8** conjugate. The characteristics of the synthesized ligand are given in Table 1. On average, one cyclodextrin torus accounts for 4-7 branched mannosylated chains of PEI.

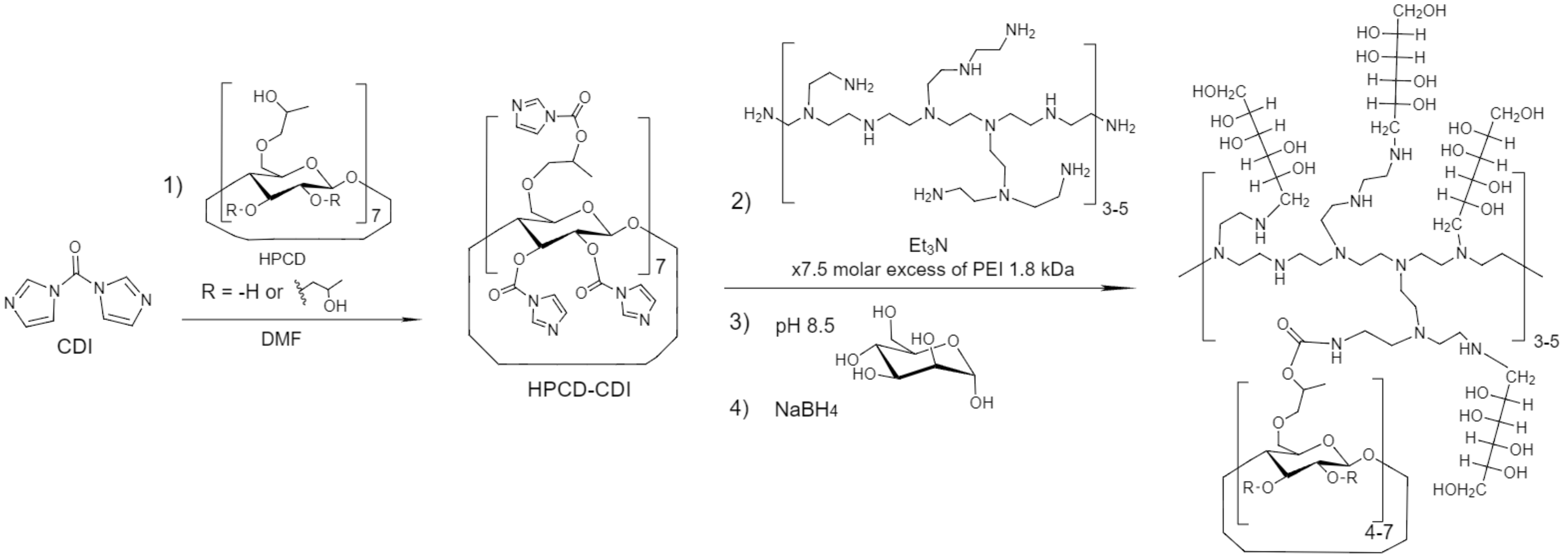

Figure S7. Decomposition of the IR spectrum of the HPCD-spermine-Man-**1** conjugate in the 980-1130 cm^{-1} region into HPCD and Man components. The fitted spectrum is given.

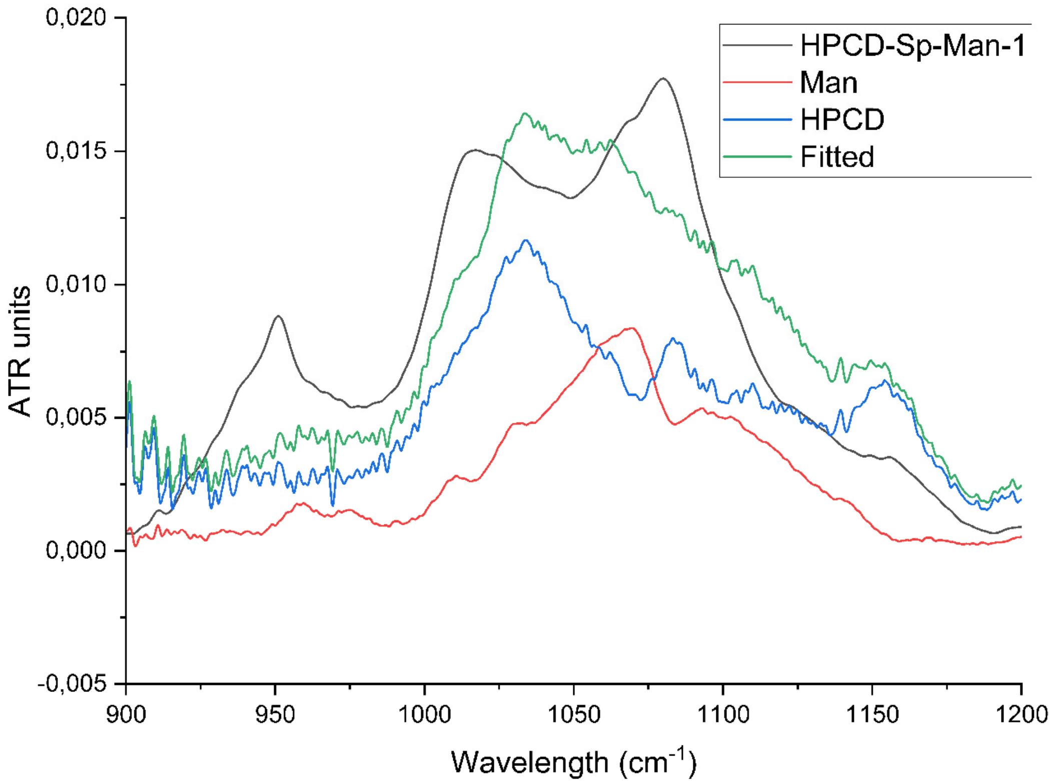

Figure S8. Fourier transform infrared spectra of mannosylated spermine-series conjugates: (a) HPCD-spermine-Man-**1**, (b) HPCD-spermine-Man-**2**, (c) HPCD-spermine-Man-**3**

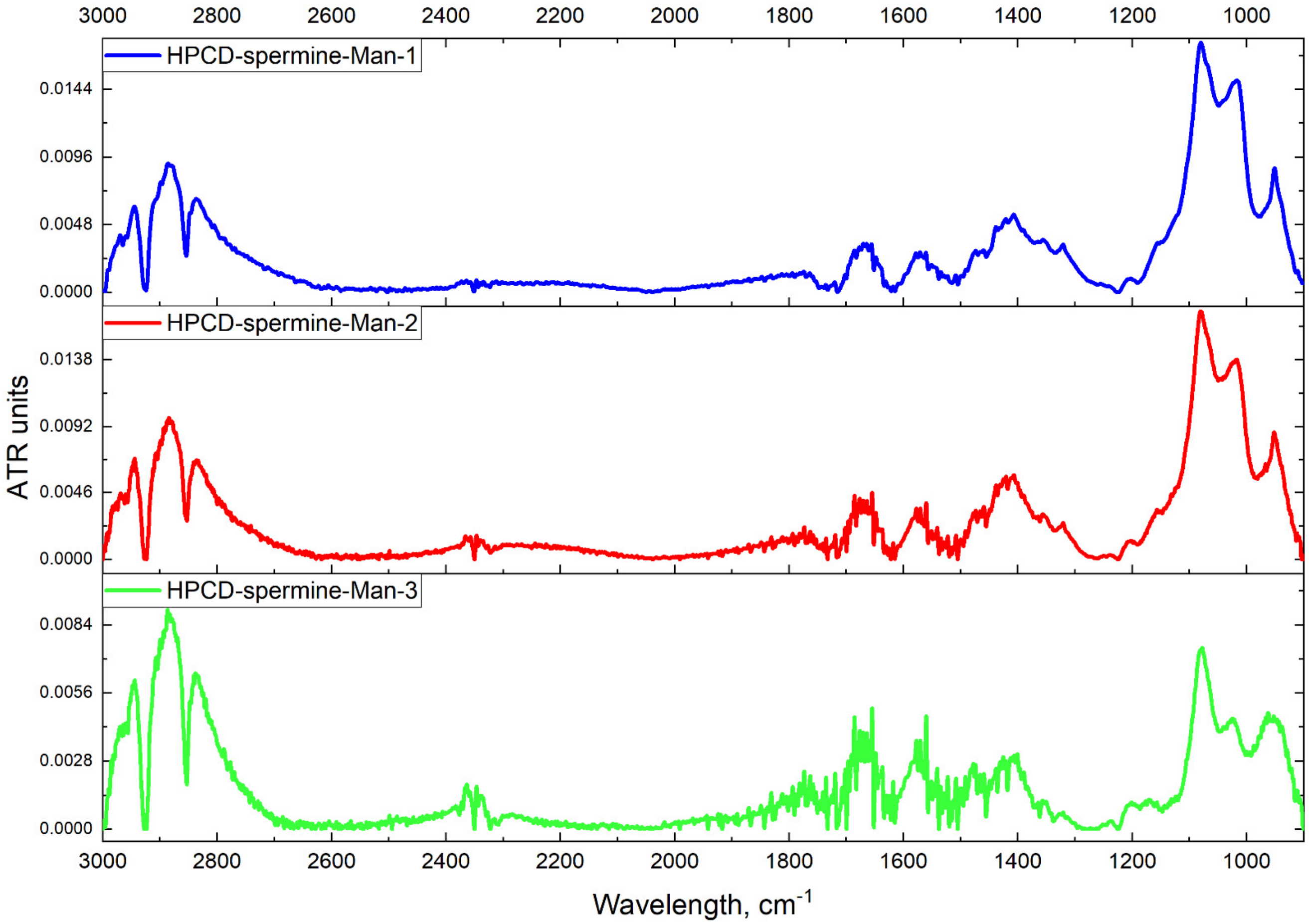

Figure S9. Fourier transform infrared spectra of mannosylated PEI35-series conjugates: (a) HPCD-PEI35-Man-**4**, (b) HPCD-PEI35-Man-**5**, (c) HPCD-PEI35-Man-**6**. (d) ^{1}H NMR spectrum of HPCD-PEI35-Man-**6**. D_2O, 12 mg/ml, 400 MHz.

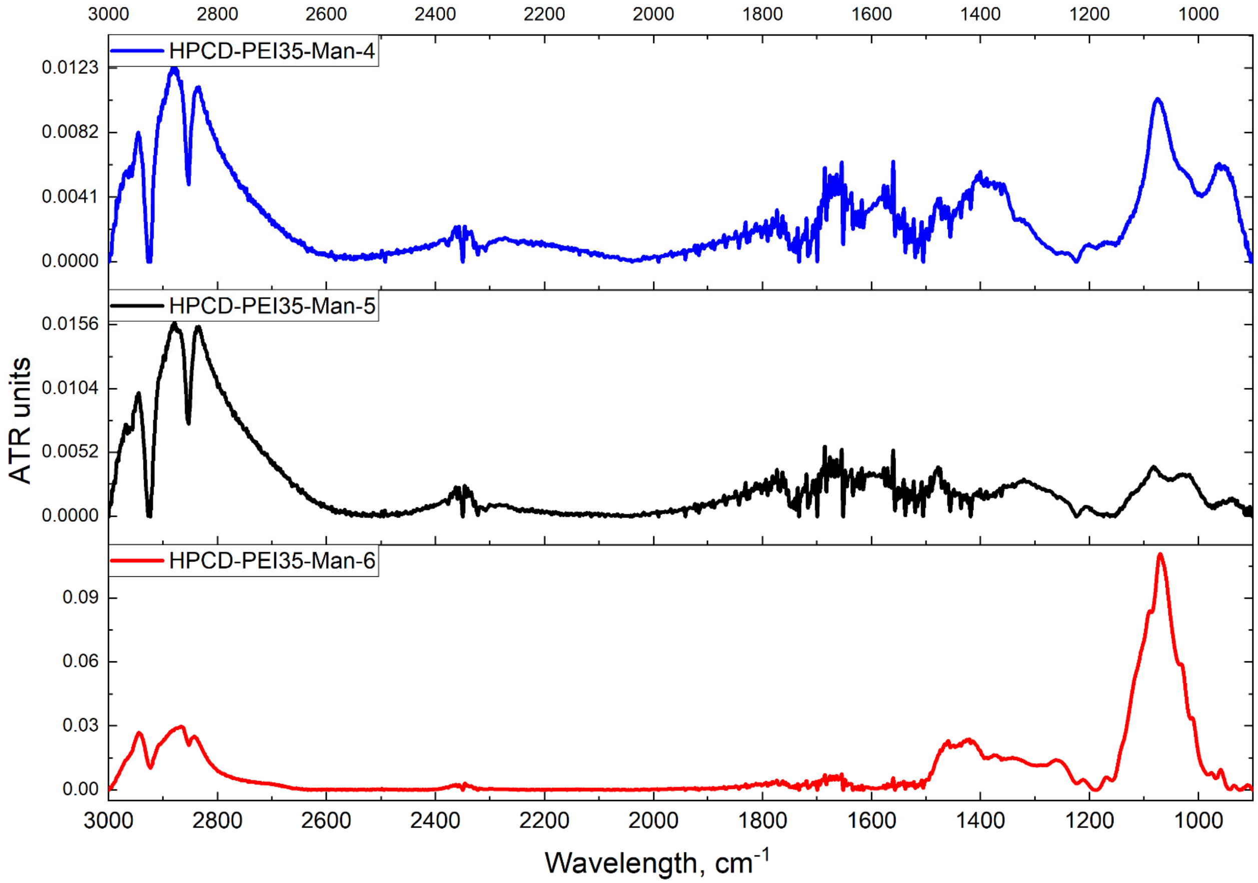

(a)–(c)

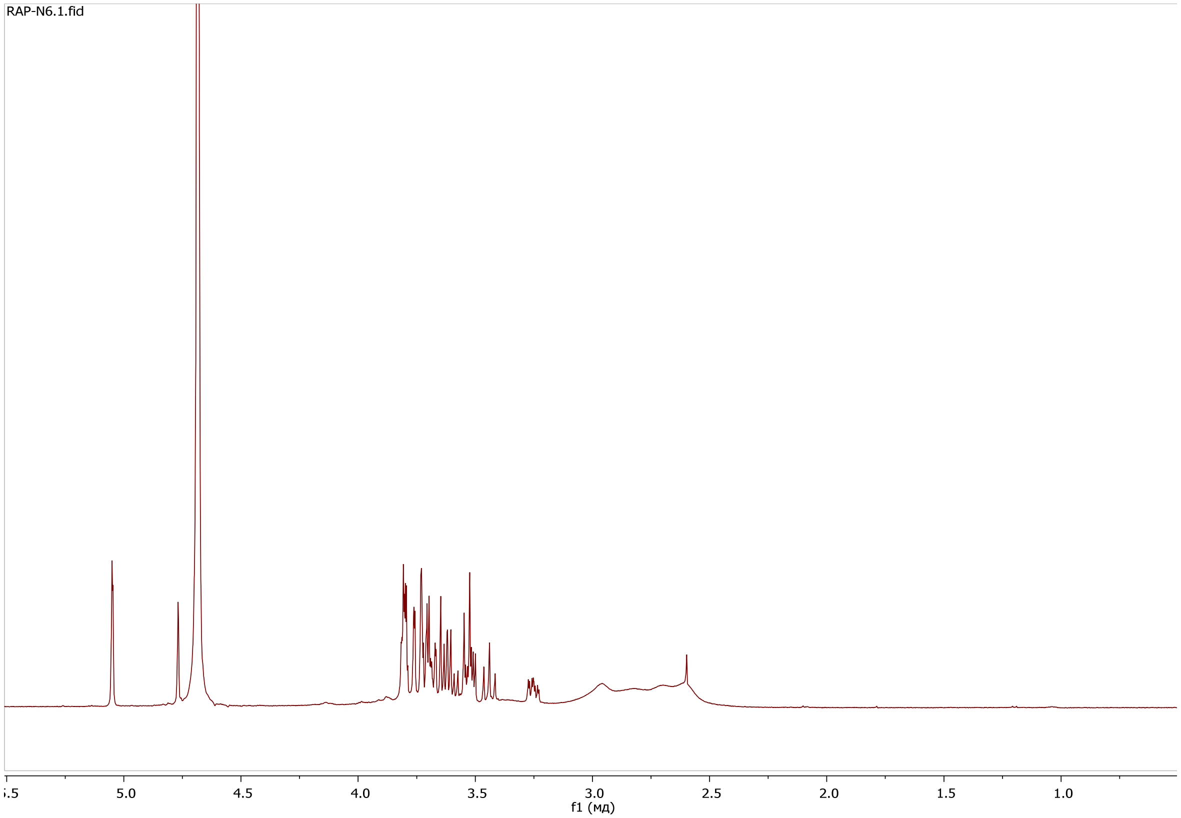

(d)

Figure S10. Fourier transform infrared spectra of mannosylated branched-PEI-series conjugates: (a) HPCD-PEI1.8-Man-**8**, (b) HPCD-PEI10-Man-**9.** ^{1}H NMR spectra of conjugates: (c) HPCD-spermine-Man-**7**, (d) HPCD-PEI1.8-Man-**8**, (e) HPCD-PEI10-Man-**9**. D_2O, 12 mg/ml, 400 MHz.

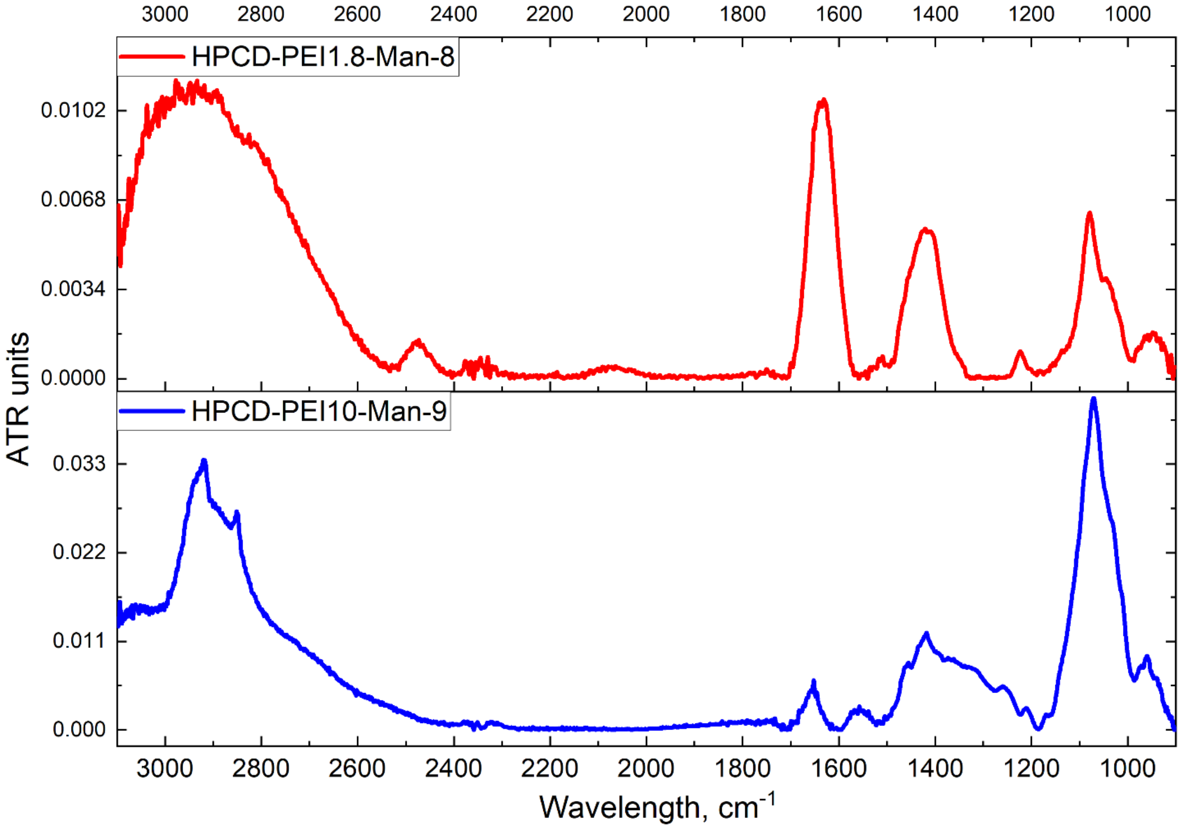

(a)–(b)

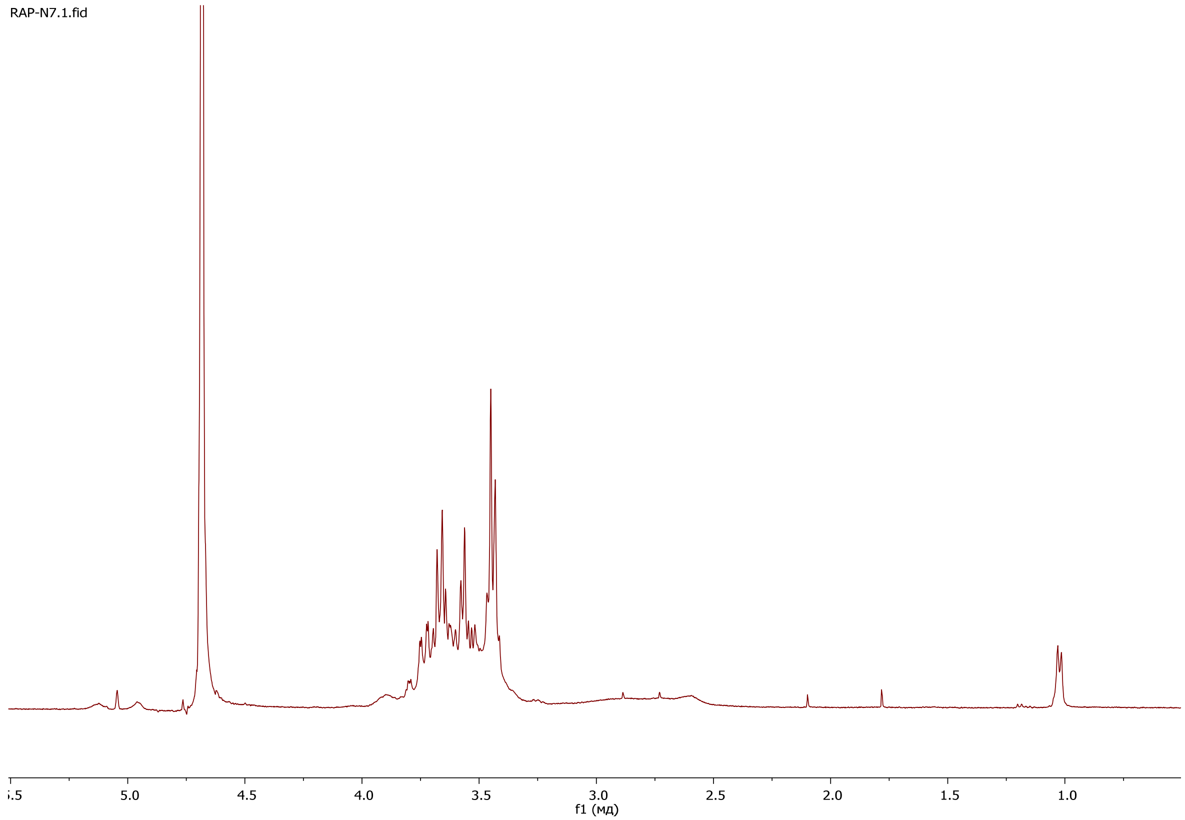

(c)

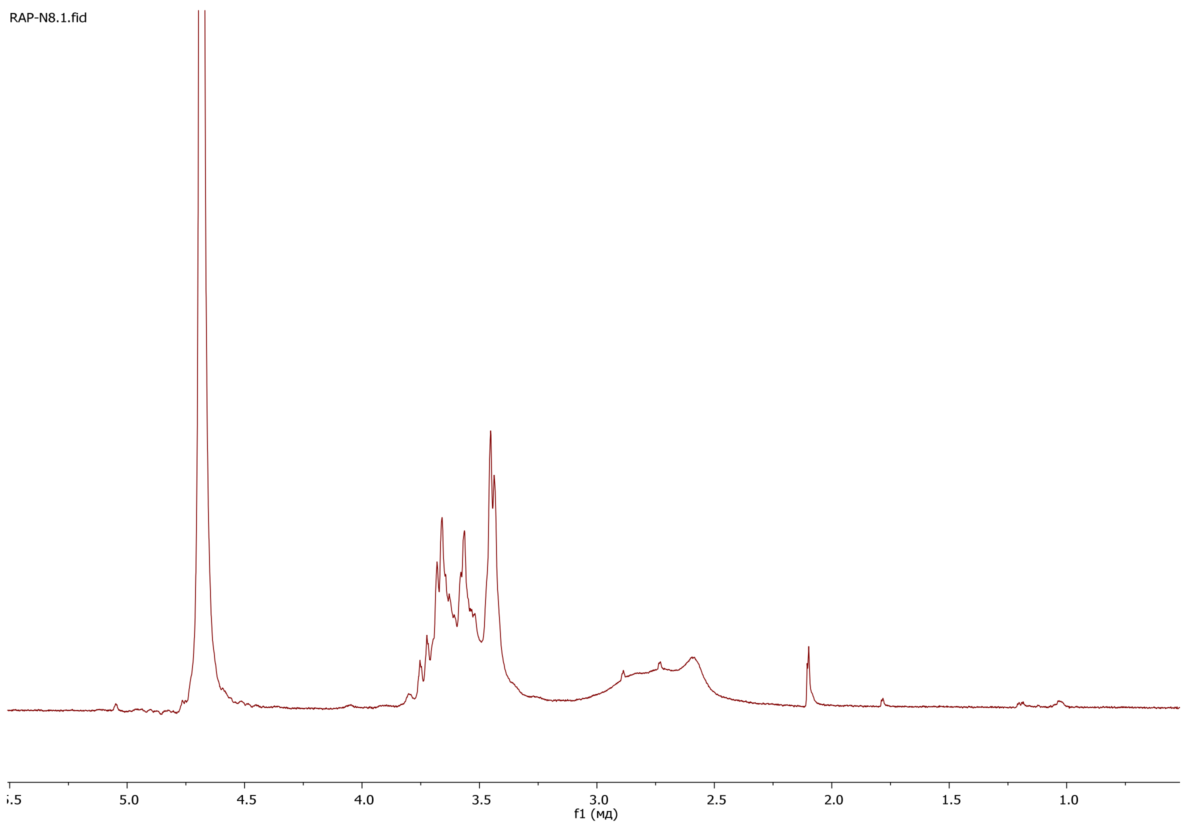

(d)

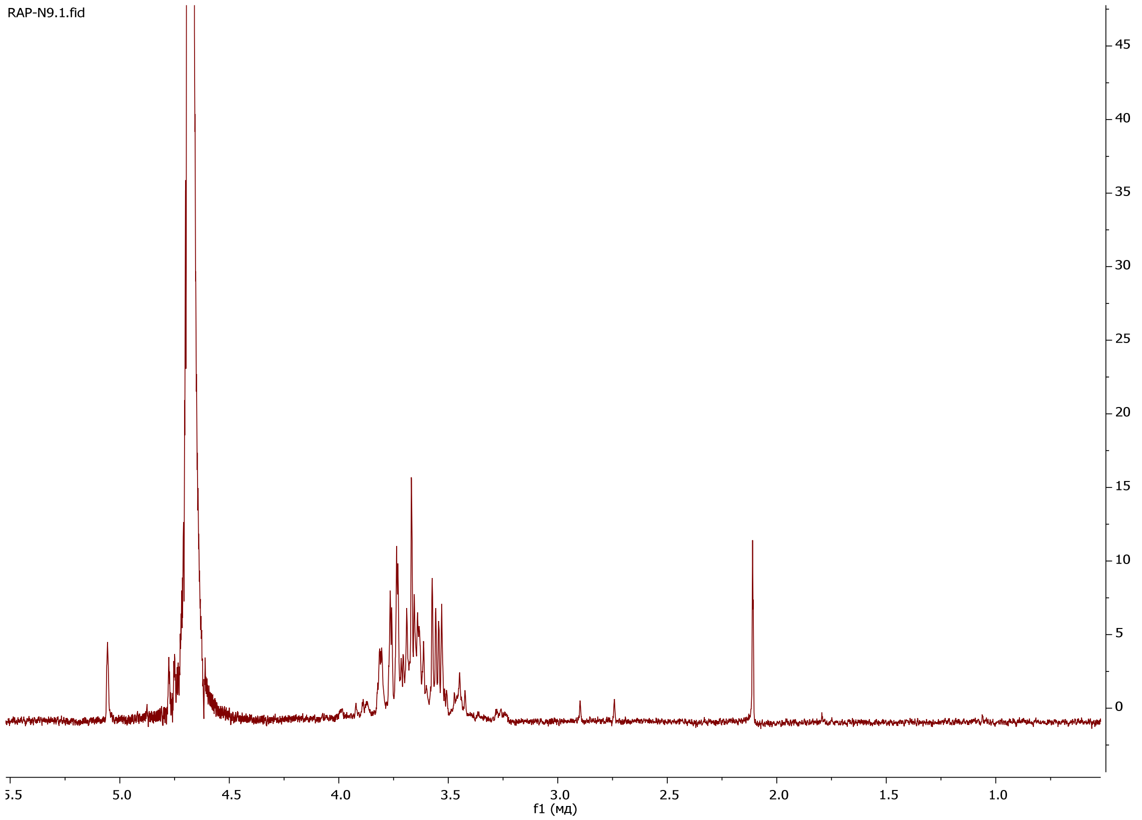

RAP-N9.1.fid
45
40
35
30
25
20
15
10
5
0
5.5
5.0
4.5
4.0
3.5
3.0
2.5
2.0
1.5
1.0
f1 (мд)

(e)

Figure S11. A fluorescent approach to studying the interaction of FITC-labeled cyclodextrin with ConA. Titration of a FITC-labeled ligand solution (C_{FITC} ≈ 0.2 µM) in 550 µl Tris/HCl buffer solution (C = 0.01 M, pH = 7.2) containing 0.5 M NaCl, 1 mM $CaCl_2$, 1 mM $MnCl_2$ was performed by adding aliquot ConA (10-25 mg/ml) 2-200 µl at 22 °C.

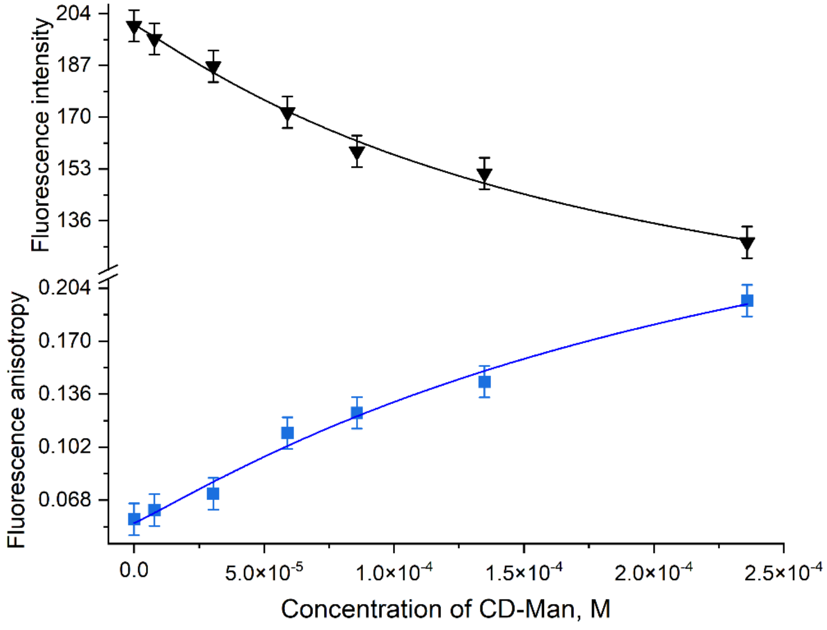

Figure S12. Stern-Vollmer's fitting plot of fluorescence intensity in case competitor binding of HPCD-PEI35-Man-**5** conjugate with ConA in mannose solution [Man] = 0.5 M. [L] – the equilibrium concentration of the ligand. C_0(dimeric ConA) = 38 μM. Ligand concentration varied from 1 to 250 μM. Natrium-citric buffer solution (0.04 M, pH 5.5). $C(Ca^{2+}) = C(Mn^{2+})$ = 1 mM. T = 22°C.

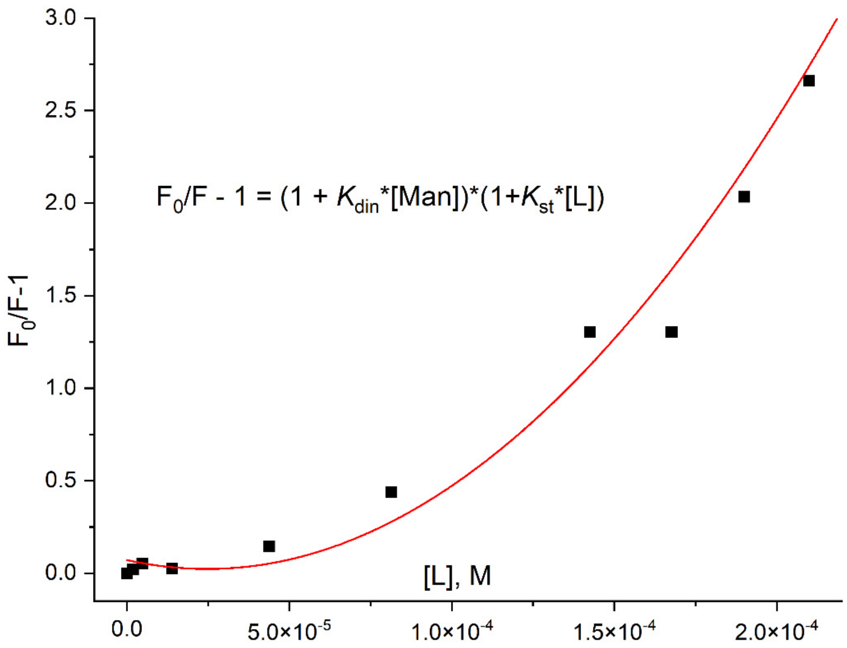

Figure S13. Tryptophan fluorescence spectra: titration of ConA by mannan. Titration of a dimeric ConA solution (C = 19 µM) in 500 µl of a sodium citrate buffer solution (C = 0.02 M, pH 5.5) containing 0.5 M NaCl, 1 mM $CaCl_2$, 1 mM $MnCl_2$ was carried out by adding aliquot of mannan at 22 °C. Concentration of mannan varied in row 0; 1.3 µM; 3.9 µM; 8 µM; 26 µM to78 µM.

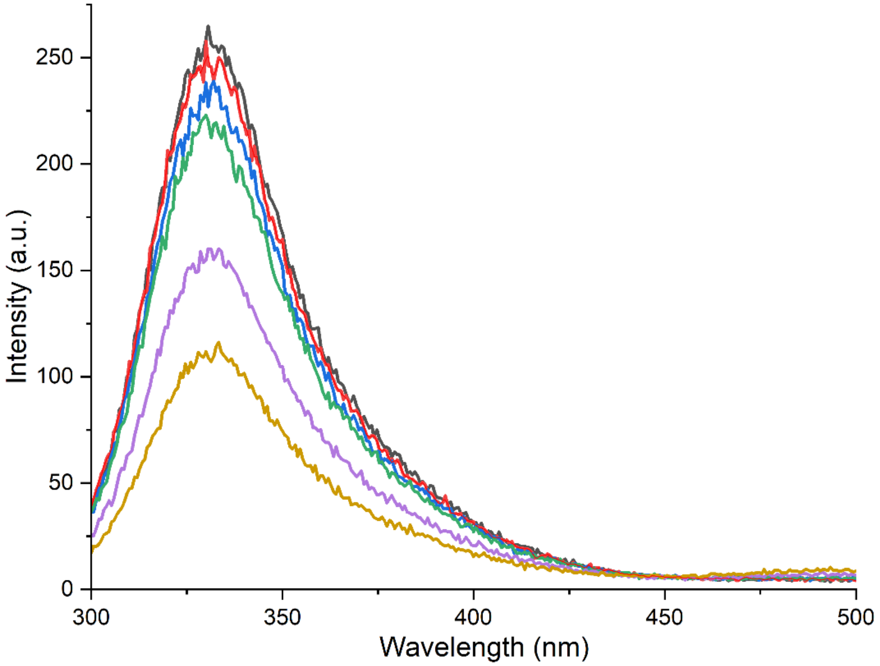

Figure S14. Complex ConA–trimannoside: X-ray diffraction data of PDB (a) and after 50-ns molecular dynamics simulation (b). The green sphere is Ca^{2+}. From [Zlotnikov 2022].

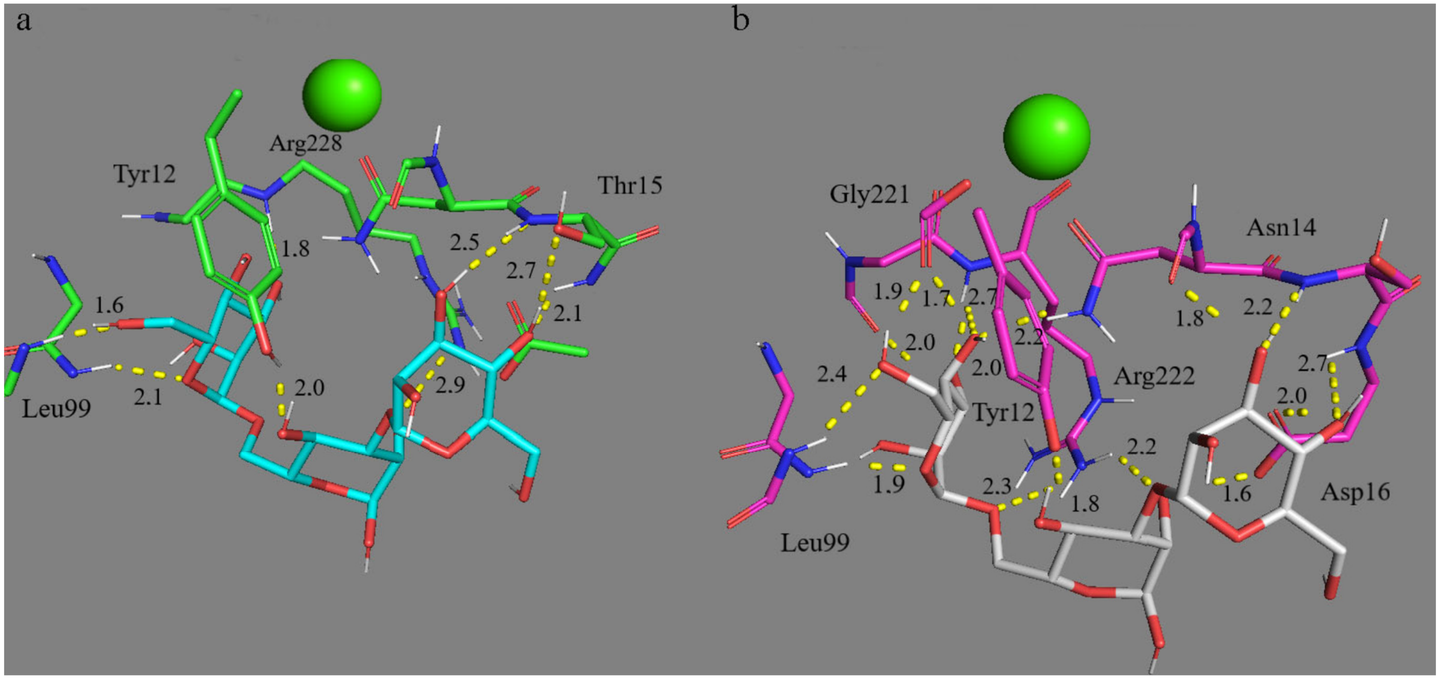

Figure S15. Maximum position in Amide II areas in differential IR spectra of the second order. Normalized to the amide I band Fourier transform infrared spectra of ConA: titration of ConA by ligand HPCD-spermine-Man-**2** and **3**. C_0(ConA) = 33 μM. Natrium-citric buffer solution (0.04 M, pH 5.5). $C(Ca^{2+}) = C(Mn^{2+})$ = 1 mM. T = 22°C.

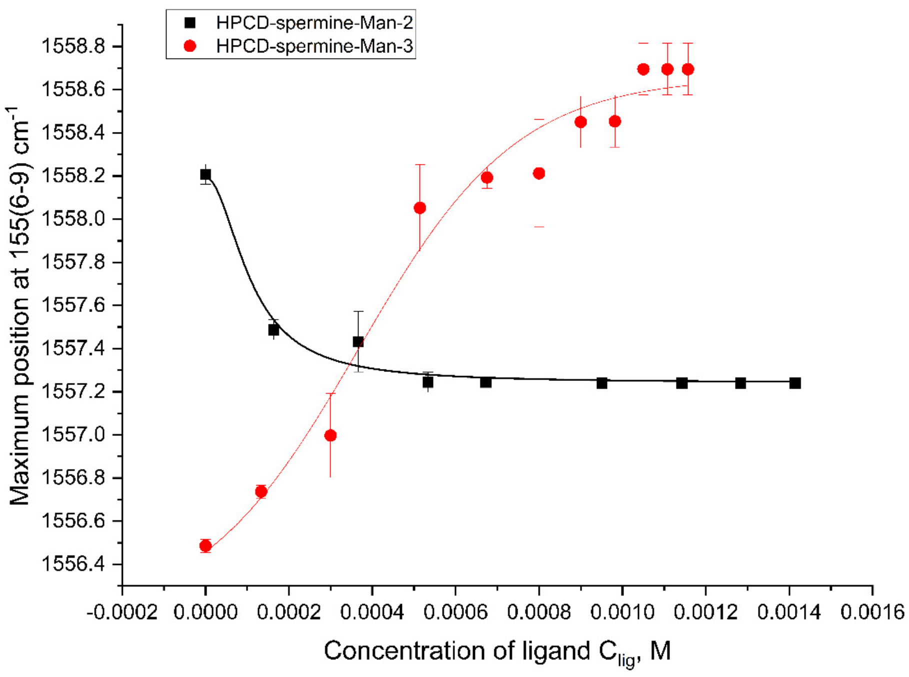

Figure S16. Hill's linearization of hyperbolic curves of dependences of maximum position in differential IR spectra of the second order (as in Figure S15). The conditions are similar to those specified in Figure S15. [L] – the equilibrium concentration of the ligand. Theta – percentage of occupied binding sites. lg K_d value is calculated as the ratio of the length of the segment cut off on the ordinate axis to the value of the tangent of the angle of inclination.

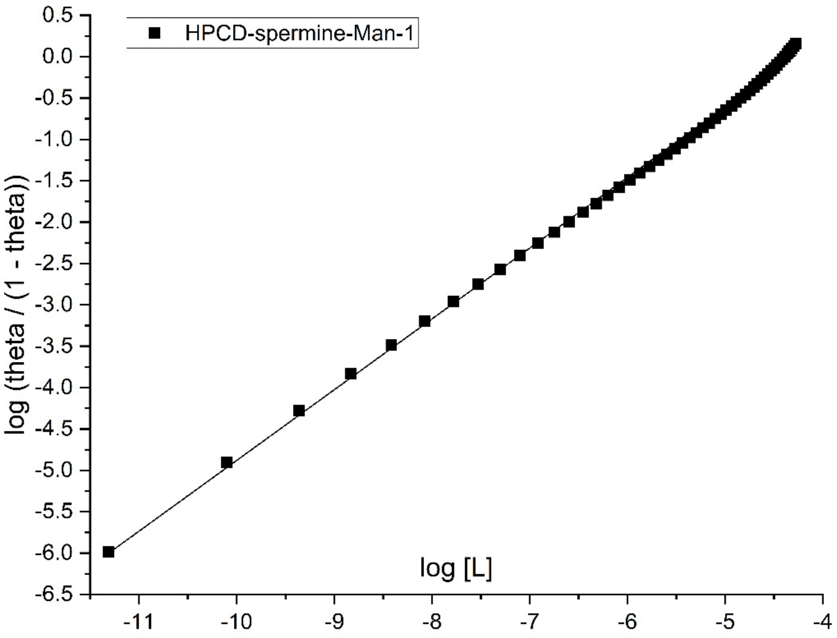

Figure S17. A fluorescent approach to studying the interaction of levofloxacin (Lev) with mesh HPCD-spermine-Man-**2** and polymer HPCD-PEI35-Man-**4** conjugates. Quenching and anisotropy of Lev fluorescence: λ_{exci} = 396 nm, λ_{emi} = 480 nm. Titration of Lev solution (C_0 = 5 mg/ml, 1 mM HCl) by ligands. T = 22°C.

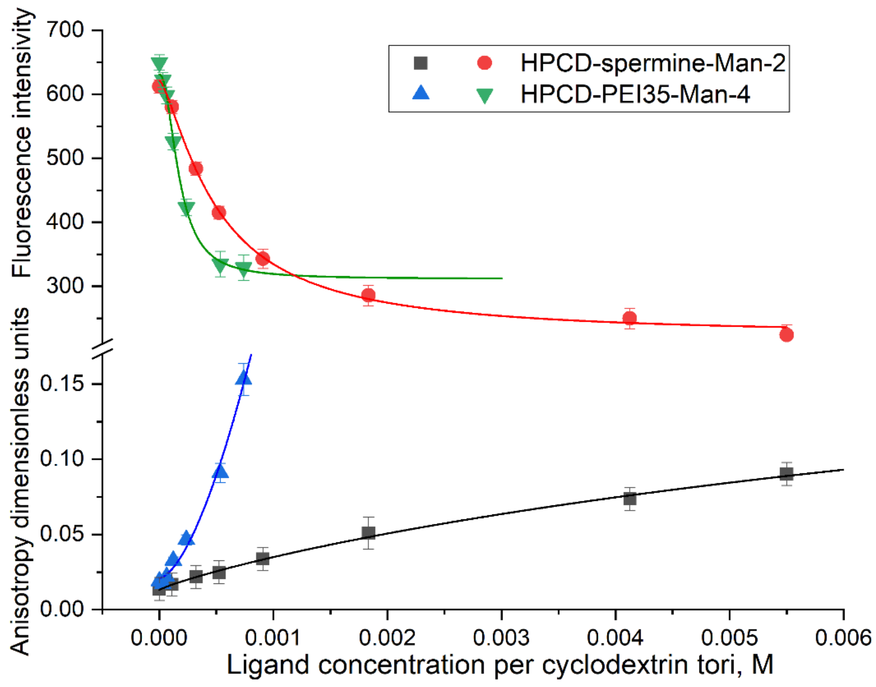

Figure S18. FTIR spectra of Lev (C_0(Lev) = 5 mg/ml) in the absence of spermine (blue spectrum) and in the presence of spermine (concentration gradient from red to green – from 2 to 5 mM). 10 mM HCl solution. T = 22°C.

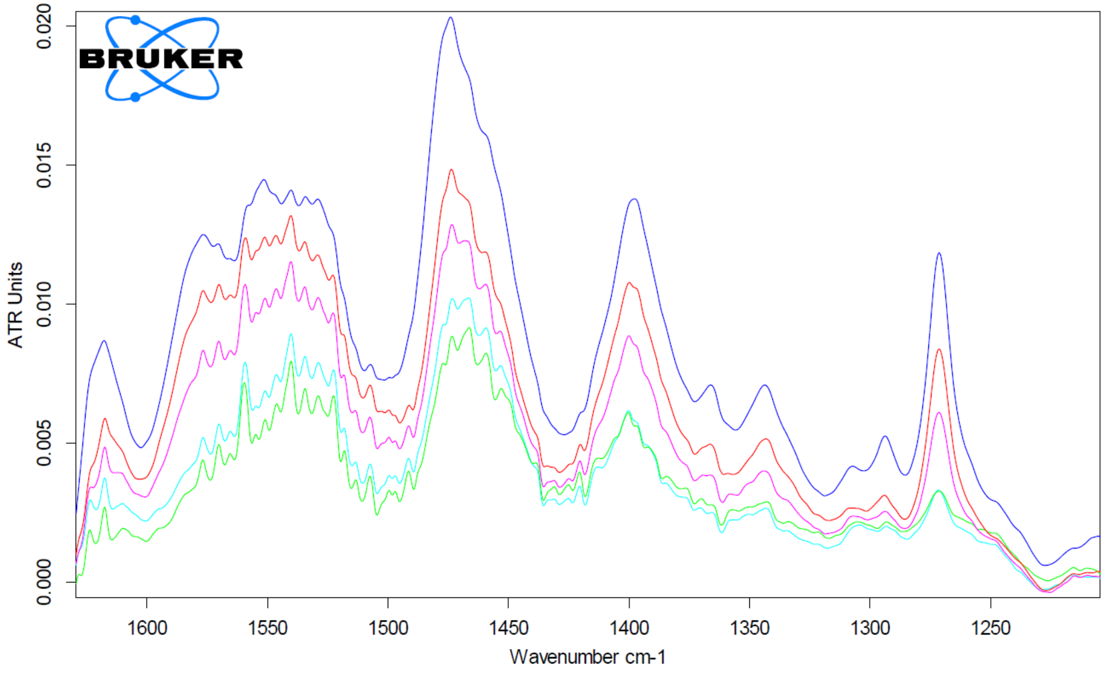

Figure S19. The dependence of the peak intensity at 1474 cm^{-1} in the IR spectra of levofloxacin complexes with conjugates normalized for the range of 2800-1800 cm^{-1}. The insert shows the normalized IR spectra for HPCD-spermine-Man-**2** complexes with Lev. C_0(Lev) = 5 mg/ml. 1 mM HCl solution. T = 22°C.

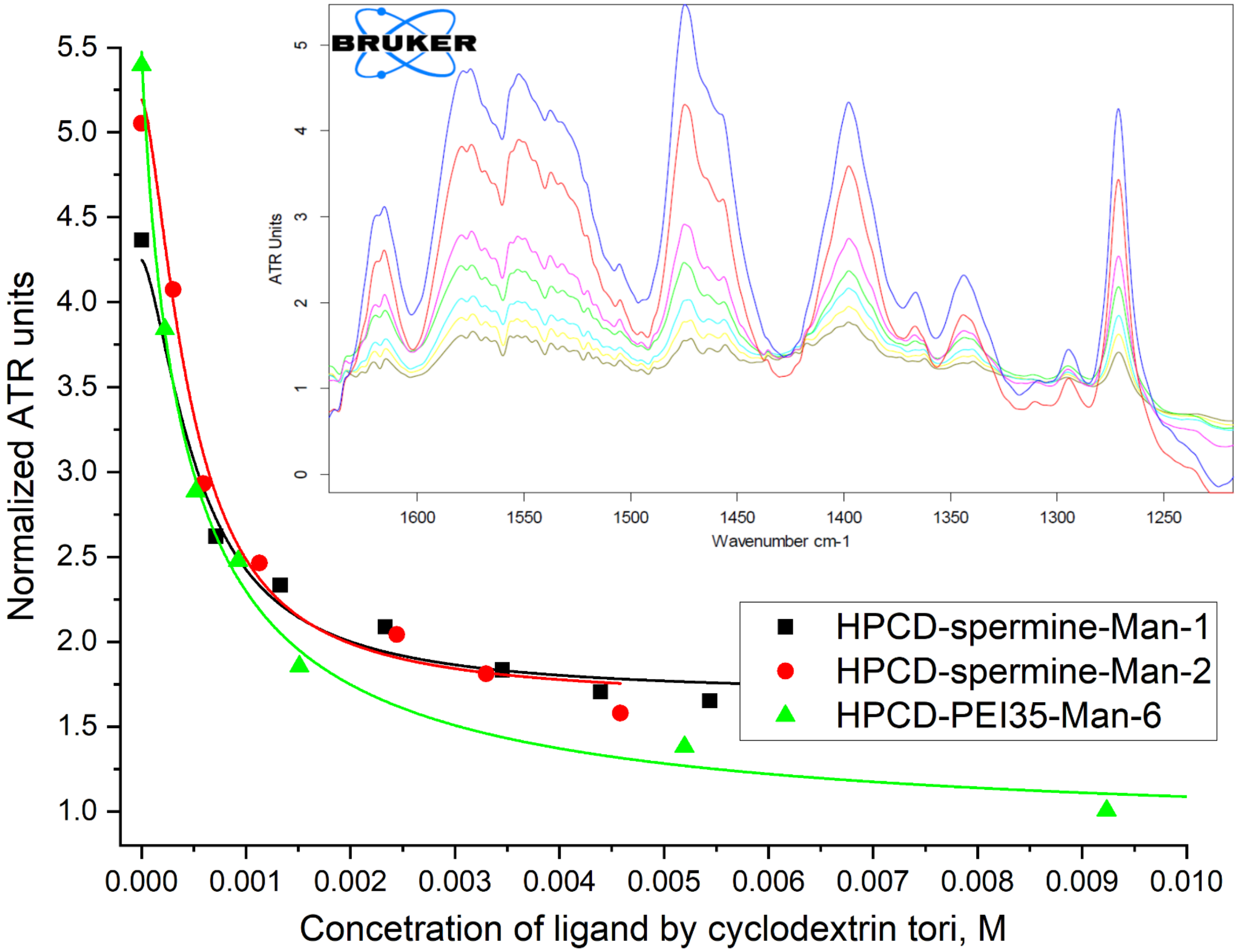

Figure S20. Solid phase FTIR spectra of levofloxacin (Lev), Lev–HPCD complex, and Lev complex with HPCD-PEI35-Man-**5** conjugate.

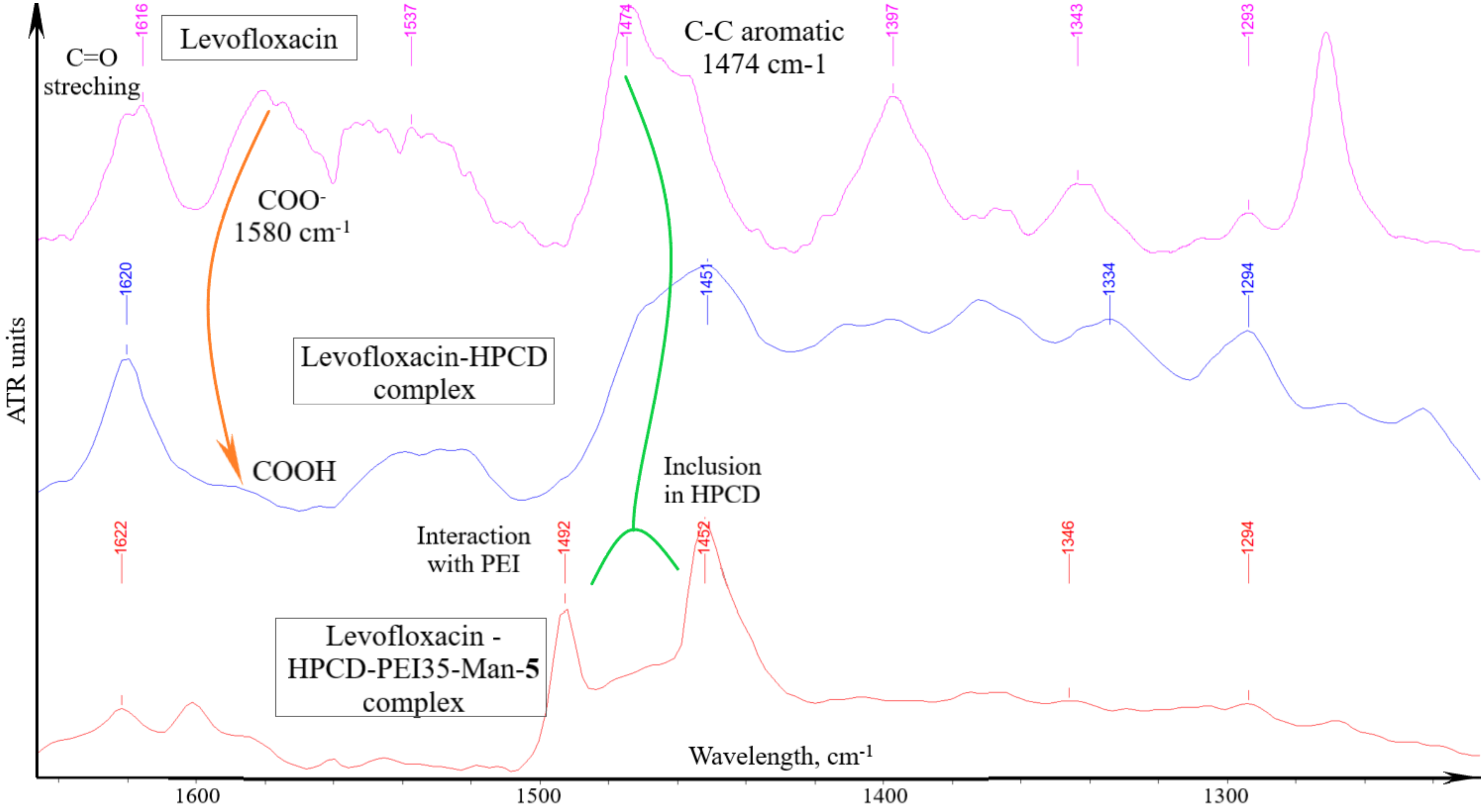

Figure S21. Solid phase FTIR spectra of eugenol, eugenol–HPCD complex, and dual (Lev ang eugenol) drug inclusion complexes with HPCD-PEI35-Man-**5** conjugate.

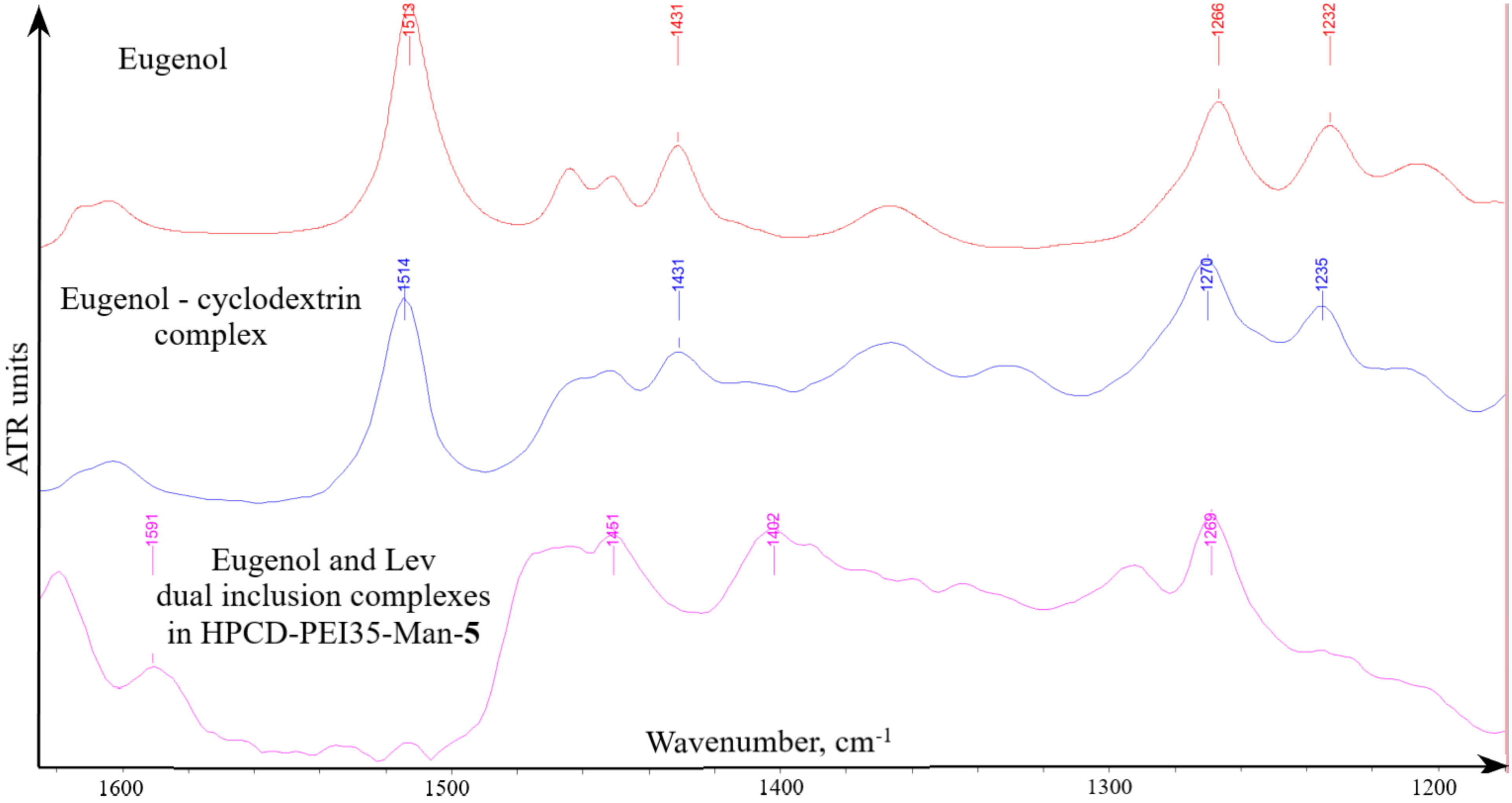

Figure S22. (a) Integral intensity of 1184–1680 cm^{-1} region in solid phase FTIR spectra of levofloxacin (Lev) and eugenol (EG) double complex with HPCD-PEI35-Man-**5** conjugate, superimposed on microphotography samples of 1x1 microns in size. (b) Corresponding FTIR spectra and areas on the micrograph (c)

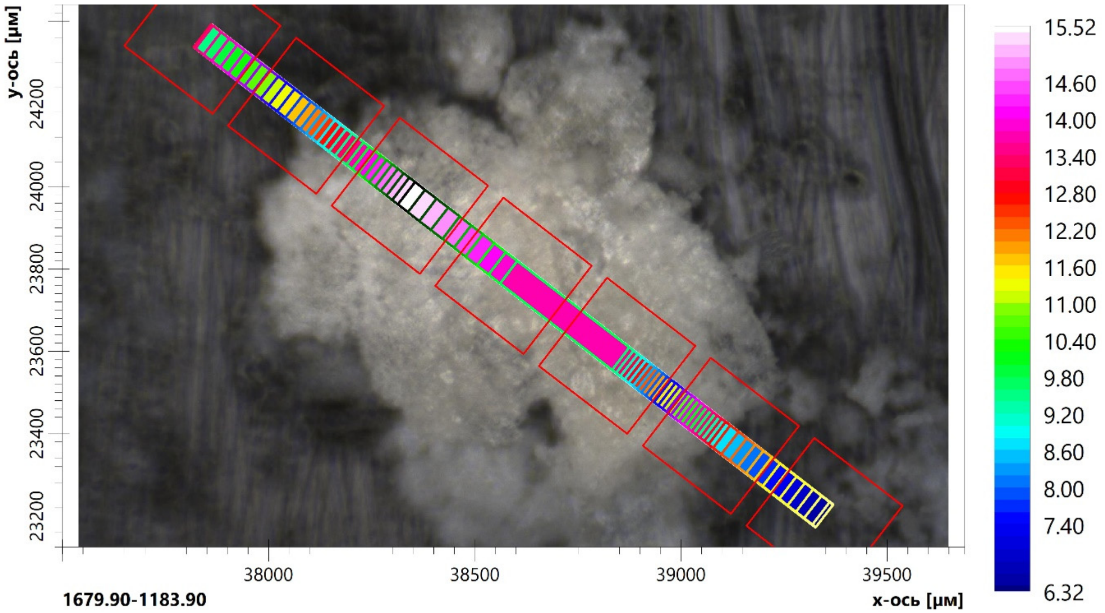

(a)

(b)

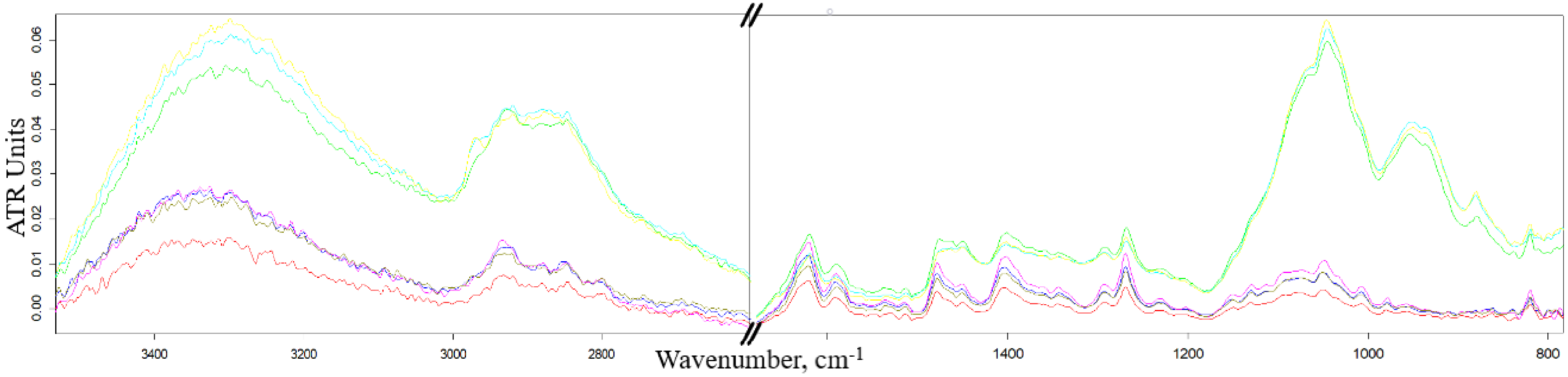

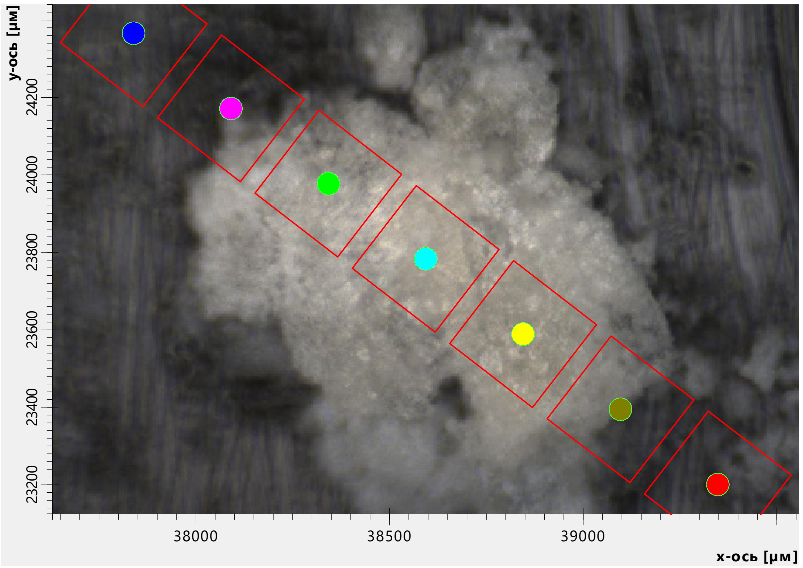
y-ось [µм]
24200
24000
23800
23600
23400
23200
38000
38500
39000
x-ось [µм]

Figure S23. Result of deconvolution of the absorption aromatic band at 1495–1415 cm^{-1} in the FTIR spectra for the (a) Lev, and (b) Lev – HPCD-PEI35-Man-**5** complex

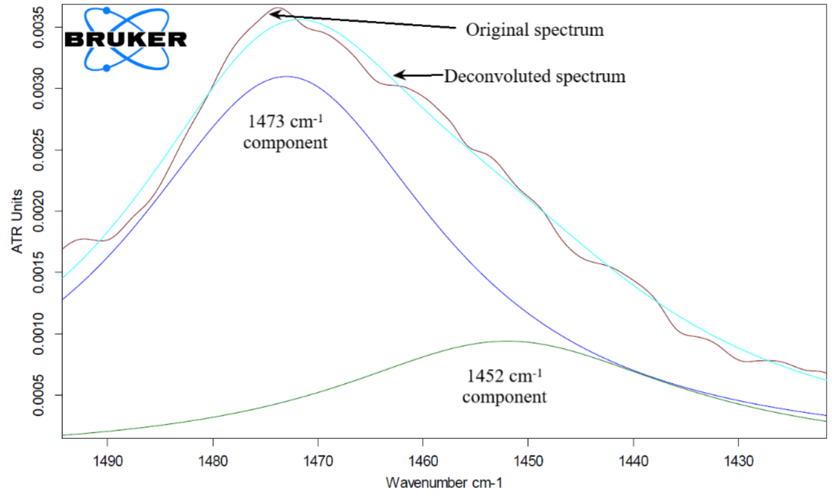

(a)

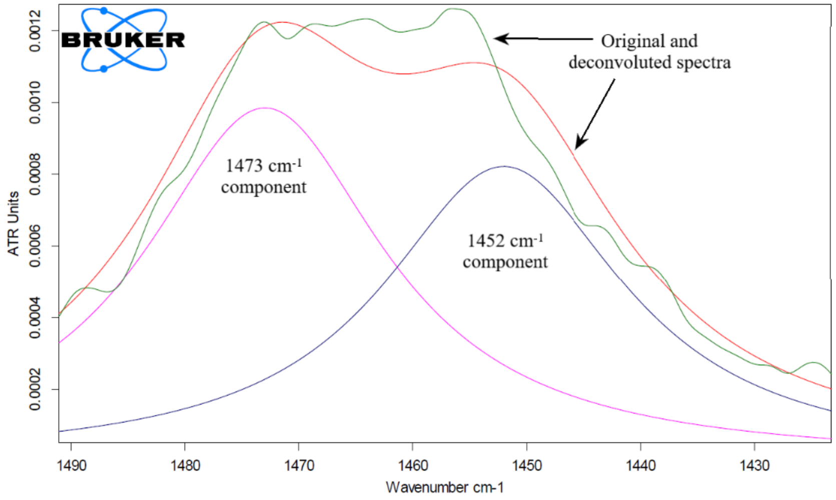

(b)

Figure S24. High-performance liquid chromatogram of conjugates HPCD-spernine-Man-**7** (5 mg/ml) and HPCD-PEI1.8-Man-**8** (two portions, 10 mg/ml). UV-absorption detector (204 nm). The eluent was 15 mM PBS (pH 7.4) containing 150 mM NaCl; the elution rate was 0.5 mL/min, 25°C.

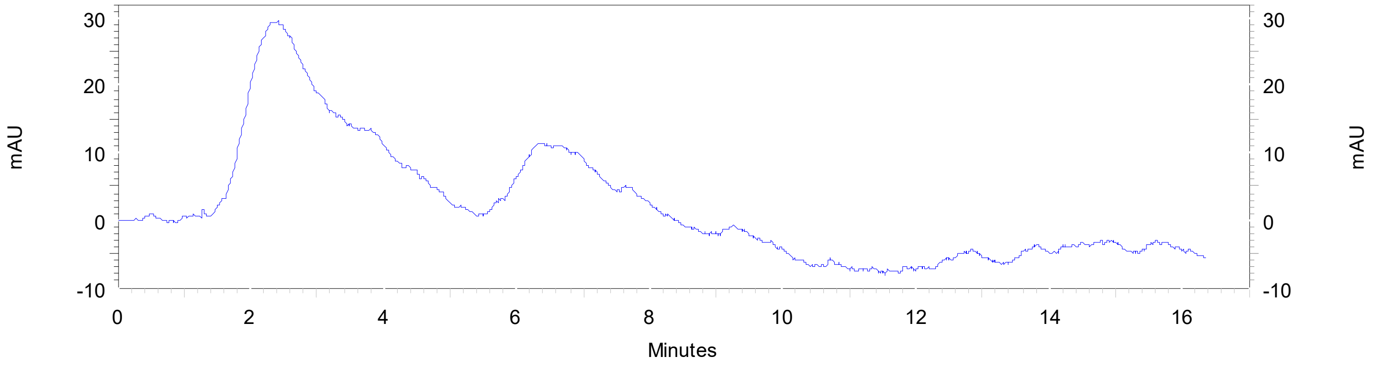

(a)

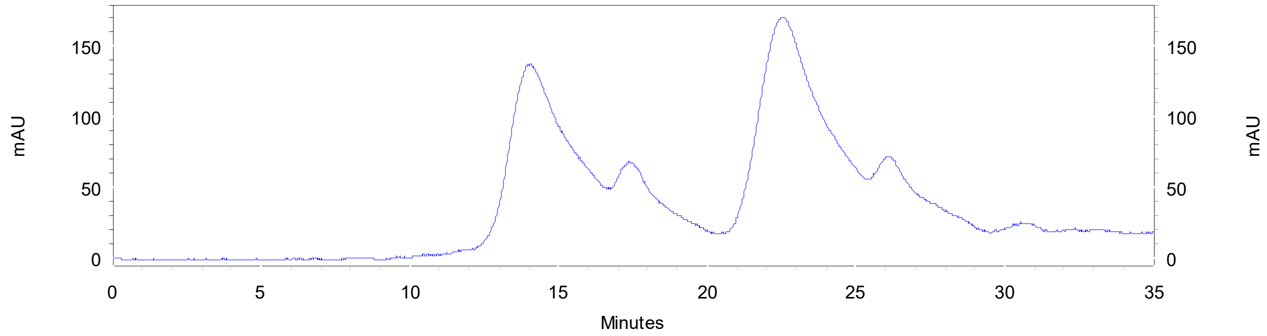

(b)

Figure S25. (a) Spectrophotometric titration of amino groups (before and after mannosylation) with 2,4,6-trinitrobenzenesulfonic acid for conjugate HPCD-spermine-Man-**7**. ε(2,4,6-trinitrobenzenesulfonic acid with NH_2-group adduct) = $1.3 \cdot 10^4$ $M^{-1} \cdot cm^{-1}$. (b) Calibration dependence of the fluorescence intensity of the reaction product on the concentration of primary amino groups (standard – spermine). Fluorescence analysis with *ortho*-phthalic aldehyde and 2-mercaptoethanol (1 minute for incubation). T = 22°C.

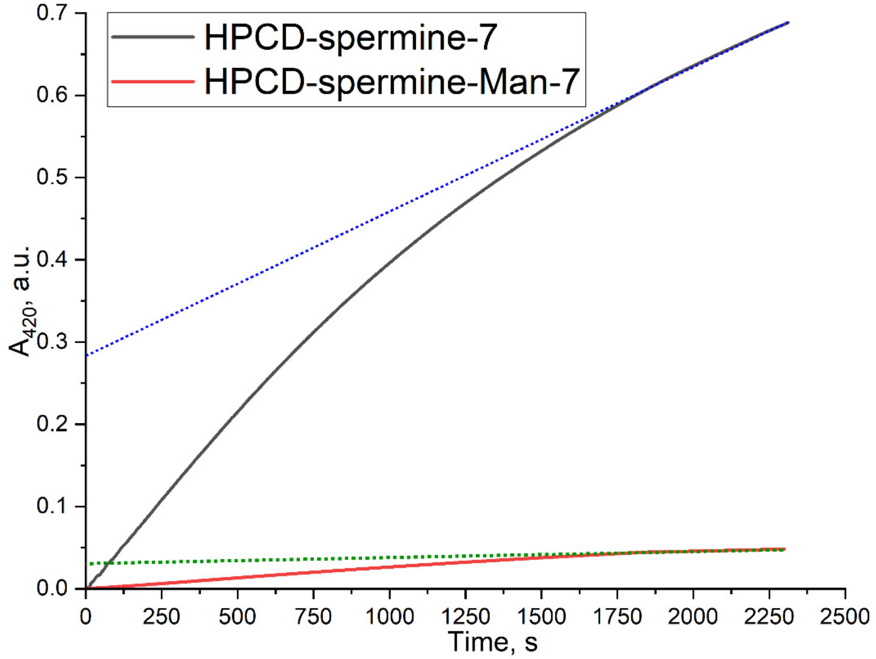

(a)

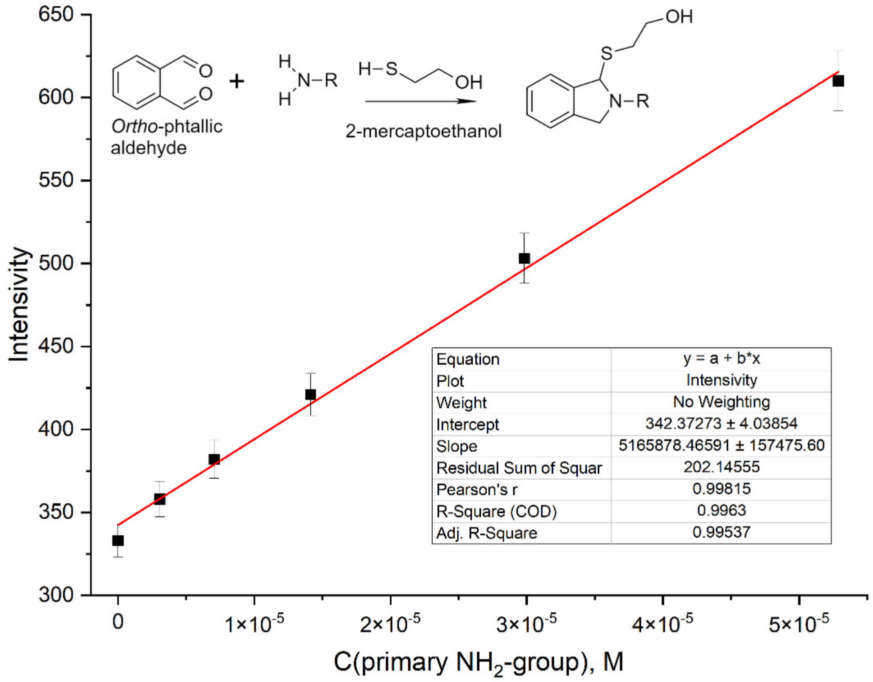

(b)
